# Pancreas as Delayed Site of Metastasis from Papillary Thyroid Carcinoma

**DOI:** 10.1155/2013/386263

**Published:** 2013-03-27

**Authors:** Mutahir A. Tunio, Mushabbab AlAsiri, Khalid Riaz, Wafa AlShakweer

**Affiliations:** ^1^Radiation Oncology, Comprehensive Cancer Center, King Fahad Medical City, Riyadh 59046, Saudi Arabia; ^2^Histopathology, Comprehensive Cancer Center, King Fahad Medical City, Riyadh 59046, Saudi Arabia

## Abstract

*Introduction*. Follicular variant (FV) papillary thyroid carcinoma (PTC) has aggressive biologic behavior as compared to classic variant (CV) of PTC and frequently metastasizes to the lungs and bones. However, metastasis to the pancreas is extremely rare manifestation of FV-PTC. To date, only 9 cases of PTC have been reported in the literature. Pancreatic metastases from PTC usually remain asymptomatic or manifest as repeated abdominal aches. Associated obstructive jaundice is rare. Prognosis is variable with reported median survival from 16 to 46 months. *Case Presentation*. Herein we present a 67-year-old Saudi woman, who developed pancreatic metastases seven years after total thyroidectomy and neck dissection followed by radioactive iodine ablation (RAI) for FV-PTC. Metastasectomy was performed by pancreaticoduodenectomy followed by sorafenib as genetic testing revealed a BRAF V600E mutation. She survived 32 months after the pancreatic metastasis diagnosis. *Conclusion*. Pancreatic metastases are rare manifestation of FV-PTC and are usually sign of extensive disease and conventional diagnostic tools may remain to reach the diagnosis.

## 1. Introduction

Thyroid cancer is the commonest endocrine malignancy, presenting with 23 500 new cases per year in the United States and European Union, respectively [1, 2]. Differentiated thyroid carcinoma (DTC) is the most frequently diagnosed cancer among women in the Middle East, behind only breast cancer, and accounting for more than 10% of all cancers among women in Saudi Arabia [3].

Papillary thyroid carcinoma (PTC) is the most frequent form of DTC and the follicular variant of PTC (FV-PTC) is more aggressive than the classic variant of PTC. It differs from classical PTC in being follicular growth pattern, higher prevalence of tumor encapsulation, angiovascular invasion, and poorly differentiated areas and a lower rate of lymph node metastases [4]. 

Pancreas is an extremely rare site of metastasis of PTC. To date, only 9 cases of pancreatic metastasis secondary to PTC have been reported in the literature. 

Herein we present a 67-year-old Saudi woman, who developed pancreatic metastasis seven years after total thyroidectomy and neck dissection followed by sorafenib for FV-PTC.

## 2. Case Presentation

**Figure 1:**
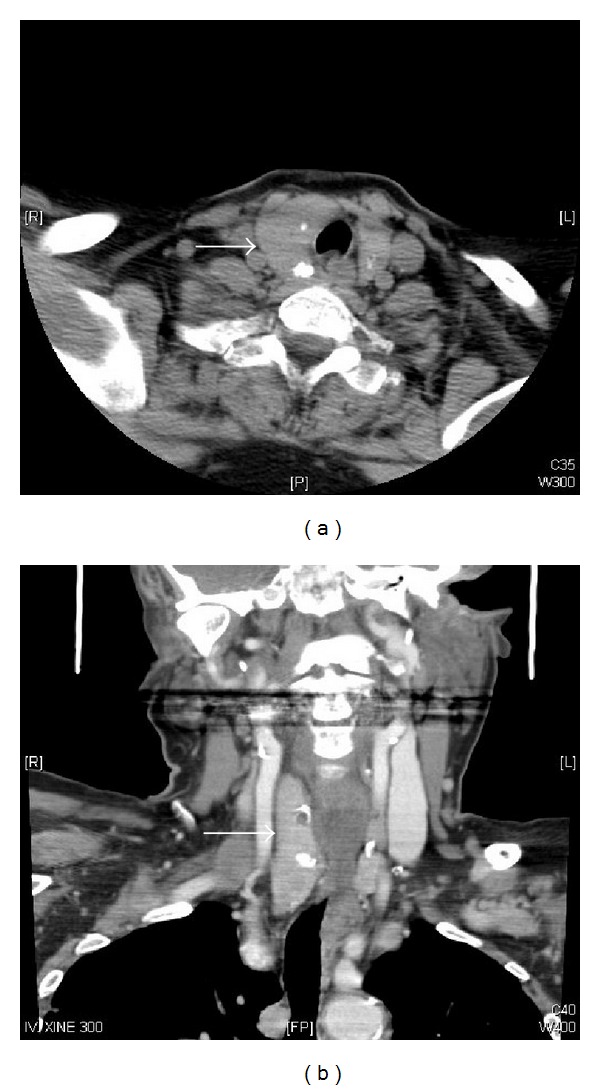
Computed tomography of neck showing diffuse enlargement of right lobe of thyroid, came out as papillary follicular cell variant thyroid carcinoma pT2N1.

In November 2009, a 67-year-old Saudi woman presented in our clinic for her routine visit with the complaints of abdominal pain and indigestion. She had noticed these complaints for 2 months and these have been occurring frequently over 2 weeks, for which she was taking antispasmodics and nonsteroidal anti-inflammatory drugs (NSAIDs), but with minimal improvement. Her previous medical history revealed hypertension and diabetes since last 20 years which were controlled on medications. She had no history of smoking and her weight was stable. Her past surgical history showed that she underwent total thyroidectomy and lymph node dissection for follicular variant papillary thyroid carcinoma pT2N1cM0 (Figure 1) in March 2002, followed by radioactive iodine (RAI) ablation 150 mCi in May 2002, and RAI ablation 150 mCi in July 2003 for raised serum thyroglobulin levels (39.3 ng/mL). 

**Figure 2:**
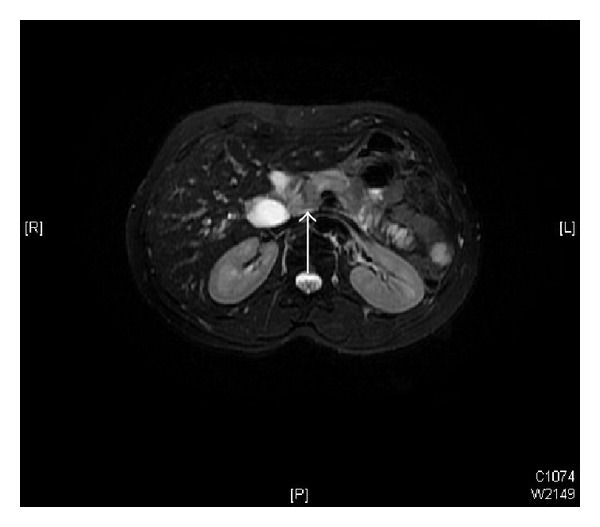
Magnetic Resonance Cholangiopancreatography (MRCP) showing a small hypovascular 1.8 × 1.5 cm mass in the pancreatic neck, invading the superior mesenteric vein.

**Figure 3:**
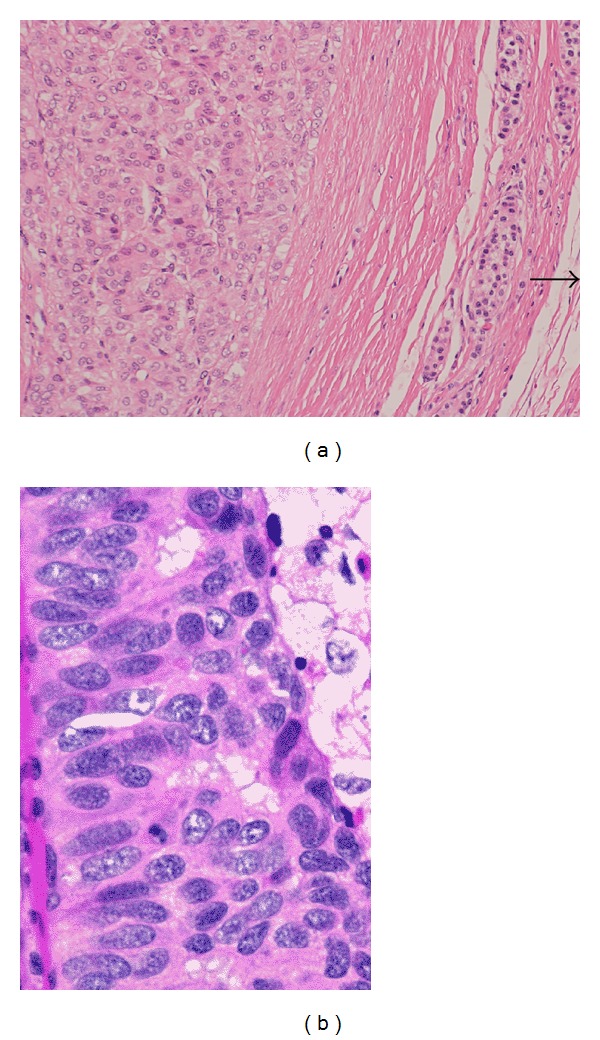
(a) Infiltrating clusters of papillary tumor cells in pancreatic tissue parenchyma (H&E ×100) and (b) follicular pattern with abundant cytoplasm (H&E ×200).

On physical examination, she was in good general condition and her vitals were stable. Per abdomen examination revealed deep tenderness in epigastrium; however, there was no palpable mass or visceromegaly. There was no other palpable cervical lymphadenopathy and examination of chest, heart, nervous system, and pelvic was normal. Clinical differential diagnosis was chronic pancreatitis, primary pancreatic adenocarcinoma, or metastasis. Her hematological, renal, and liver function tests, tuberculin, serum electrolytes, and thyroid stimulating hormone (TSH) and thyroxin (T4) were found within normal limits. Serum tumor markers, carcinoembryonic antigen (CEA), and cancer antigen 19-9 were also normal; however, serum TG levels were raised, that is, 672.8 ng/mL (normal: 5–25 ng/mL). Computed tomography (CT) of abdomen showed decreased enhancement of the neck, body, and tail of the pancreas; while enhancement of the pancreatic head appeared normal and there were bilateral multiple pulmonary metastases. Whole body iodine scintigraphy was noniodine avid. Magnetic Resonance Cholangiopancreatography (MRCP) revealed a small hypovascular lesion in the pancreatic neck measuring 1.8 × 1.5 cm. It showed low signal intensity in both T1 and T2 with no enhancement in the arterial phase and faint enhancement in delayed sequences. Mass was abutting the superior mesenteric vein (SMV) and caused narrowing to its caliber below the confluence with splenic vein; however, superior mesenteric artery (SMA) was found normal in caliber with no sign of invasion (Figure 2). CT guided fine needle aspiration cytology of the mass was performed and it showed papillary nests of eosinophilic tumor cells with intranuclear inclusions. The patient underwent pancreaticoduodenectomy. Histopathology showed 1.6 × 1.4 cm metastatic deposit in the neck of the pancreas and immunohistochemistry examination showed the positivity for TG and thyroid transcription factor-1 (TTF-1) and made confirmed diagnosis of pancreatic metastasis consistent with FV-PTC (Figure 3). Genetic testing revealed a BRAF V600E mutation. After surgical resection, she was started on sorafenib 400 mg twice daily, which she tolerated well. At time of submission of case report the patient was alive at 36 months postoperatively and was doing fine with partial response in lungs, without recurrence in neck and serum TG levels 39.8 ng/mL. 

## 3. Discussion

Pancreas is rare site metastasis. Common malignancies which metastasize to pancreas are renal cell carcinoma, lung, medullary carcinoma of the thyroid, lymphomas, alveolar rhabdomyosarcoma, and esophagus [11]. Metastases to the pancreas from papillary thyroid carcinoma are extremely rare. To date, only 9 cases have been reported in the literature with initial diagnosis of PTC [5–10, 12] in Table 1. Exact pathogenesis is not well known; however, hematogenous route is well supported.

Pancreatic metastases remain asymptomatic for long period before diagnosis or manifest as symptoms like chronic pancreatitis as seen in our patient. At time of presentation, pancreatic metastases are usually sign of extensive disease seen in our patient. Conventional diagnostic tools (CT, WBS) may remain fail to reach the diagnosis. However, positron emission tomography-computed tomography (PET-CT) and endoscopic ultrasound (EUS) have shown the sensitivity or detecting pancreatic lesions up to 94–100%, though not performed in our case due to unavailability in institute [13]. 

Surgical treatment in the form of pylorus sparing pancreaticoduodenectomy relieves the symptoms and may increase the disease free survival (DFS) for isolated pancreatic metastasis. However, adjunct and multikinase inhibitors (sorafenib or sunitinib) may slow the disease progression especially in patients with BRAF V600E mutation in tumor cell lines [14]. 

In conclusion, pancreatic metastases are rare in FV-PTC and they remain asymptomatic or are associated with nonspecific complaints. Improved diagnostic tools (MRCP, PET-CT, and EUS) and immunohistochemistry can be helpful for prompt diagnosis and treatment by means of pancreaticoduodenectomy followed by sorafenib which is associated with increased DFS.

## Figures and Tables

**Table 1:** Cases of pancreatic metastasis secondary to papillary thyroid carcinoma reported from 1991 to 2012.

Author	Age	From time of initial treatment	Variant	Treatment	Survival after diagnosis of pancreatic metastasis
Zhu et al. [4]	NA	NA	Tall cell	Surgical resection	NA

Sugimura et al. [5]	53 years	7 years after TT + RAI	Classical	Surgical resection	NA

Jobran et al. [6]	53 years male	NA	Tall cell	Surgical resection	NA

Angeles-Angeles et al. [7]	72 years male	NA	Classical	Surgical resection	NA

Borschitz et al. [8]	34 years female	9 years after TT + RAI	Classical	Surgical resection	42 months
	46 years male	2 years after TT + RAI	Follicular		

Chen and Brainard [9]	82 years male	5 years after TT + RAI	Classical	Surgical resection	NA

Alzahrani et al. [10]	56 years male	6 years after TT + RAI	Classical	Sorafenib	20 months

Present case	67 years	7 years after TT + RAI	Tall cell	Surgical resection and RAI	Alive at 32 months

TT: total thyroidectomy, RAI: radioactive iodine therapy, NA: not mentioned.
